# *Acinetobacter tandoii* ZM06 Assists *Glutamicibacter nicotianae* ZM05 in Resisting Cadmium Pressure to Preserve Dipropyl Phthalate Biodegradation

**DOI:** 10.3390/microorganisms9071417

**Published:** 2021-06-30

**Authors:** Xuejun Wang, Si Shen, Hao Wu, Haixia Wang, Lvjing Wang, Zhenmei Lu

**Affiliations:** MOE Laboratory of Biosystem Homeostasis and Protection, College of Life Sciences, Zhejiang University, Hangzhou 310058, China; wxj941031@163.com (X.W.); sshen10@its.jnj.com (S.S.); wuhaochs@hotmail.com (H.W.); lanedysia@hotmail.com (H.W.); wanglvjing@zju.edu.cn (L.W.)

**Keywords:** metatranscriptomics, *Glutamicibacter nicotianae* ZM05, *Acinetobacter tandoii* ZM06, cooperation, DPrP biodegradation, cadmium pressure

## Abstract

Dipropyl phthalate (DPrP) coexists with cadmium as cocontaminants in environmental media. A coculture system including the DPrP-degrading bacterium *Glutamicibacter nicotianae* ZM05 and the nondegrading bacterium *Acinetobacter tandoii* ZM06 was artificially established to degrade DPrP under Cd(II) stress. Strain ZM06 relieved the pressure of cadmium on strain ZM05 and accelerated DPrP degradation in the following three ways: first, strain ZM06 adsorbed Cd(II) on the cell surface (as observed by scanning electron microscopy) to decrease the concentration of Cd(II) in the coculture system; second, the downstream metabolites of ZM05 were utilized by strain ZM06 to reduce metabolite inhibition; and third, strain ZM06 supplied amino acids and fatty acids to strain ZM05 to relieve stress during DPrP degradation, which was demonstrated by comparative transcriptomic analysis. This study provides an elementary understanding of how microbial consortia improve the degradation efficiency of organic pollutants under heavy metals contamination.

## 1. Introduction

Phthalic Acid Esters (PAEs), as plasticizers, can migrate out from plastic materials into the environment during their production and disposal [1]. Dipropyl phthalate (DPrP), as a kind of PAEs with mutagenic, teratogenic, and carcinogenic properties, has been classified as a priority pollutant by the United States Environmental Protection Agency [2]. Furthermore, it has recently been proven that DPrP causes cacoethic effects on human health by interfering with endogenous hormones [3].

In environments such as soil and groundwater, PAEs often cocontaminate with heavy metals. Compared with that of individual contamination, the remediation of cocontamination with organic pollutants and heavy metals is a more complicated problem owing to the combined toxicity and potential interactions of contaminants [4,5]. Cadmium (Cd) and PAEs are of particular concern due to their migration and bioaccumulation [6], potentially harmful effects on the ecosystem and human health, and frequent occurrence in several types of anthropogenic contaminated sites. Agricultural production with cadmium content in fertilizer applications and atmospheric cadmium dust settlement directly causes the copollution of cadmium and PAEs [7,8]. A large body of evidence actually shows that PAEs and heavy metals coexist in the environment [9,10,11].

Microorganisms play crucial roles in biogeochemical cycling in ecosystems [12] and may mediate metal detoxification [13] and the biodegradation of organic compounds [14]. However, previous studies have shown that heavy metals have an adverse impact on the microbial degradation of organic pollutants by repressing microbial activity [12]. Hence, for the remediation of cocontamination with organic compounds and heavy metals, microbial consortia exhibit superior biodegradation performance [15,16]. Compared with a single strain, bacterial consortia can facilitate the degradation of organic pollutants by improving environmental adaptability [17], alleviating environmental stress [15], and cooperating with metabolite-degrading bacteria [18]. For example, the cadmium-resistant bacterium *Pseudomonas* sp. H1 enhanced the degradation of 2,4-dichlorophenoxyacetic acid by *Ralstonia eutropha* JMP134 in the presence of 60 μg/g cadmium by reducing the stress [19]. Interactions between microorganisms play important roles in the degradation of hazardous materials. Therefore, constructing bacterial consortia that resist heavy metals and degrade organic pollutants is an ideal solution for remediating combined contamination, and it is essential to explore the interaction mechanisms of microbial cooperative degradation during remediating cocontamination.

Based on a previous study of ours, an efficient PAEs-degrading bacterium was identified as *Glutamicibacter nicotianae* ZM05, which could degrade most short-chain PAEs, including dibutyl phthalate (DBP), DPrP, diethyl phthalate (DEP), and dimethyl phthalate (DMP) [20]. Nevertheless, the growth and degradation ability of strain ZM05 were depressed under heavy metal contamination, especially cadmium pollution. In this study, an artificially constructed consortium composed of the DPrP-degrading bacterium ZM05 and the nondegrading bacterium *Acinetobacter tandoii* ZM06 effectively resisted the inhibition of DPrP degradation by Cd(II) stress. To study the cooperative interactions during degradation strains ZM05 and ZM06 were cocultured under Cd(II) stress or without Cd(II) stress and subjected to metatranscriptomics. We centered on the differences in DPrP degradation and gene expression in the degrading bacterium ZM05 between the monoculture and coculture systems with and without Cd(II) stress.

## 2. Materials and Methods

### 2.1. Chemicals and Media

Di-n-propyl phthalate (DPrP, >98% purity), diethyl phthalate (DEP, >99% purity), dimethyl phthalate (DMP, >99% purity), monomethyl phthalate (MMP, >97% purity), and phthalic acid (PA, >99.5% purity) were purchased from Aladdin Industrial Corporation (Shanghai, China), while monoethyl phthalate (MEP, >98% purity) was purchased from Solarbio Science & Technology Co., Ltd. (Beijing, China), and protocatechuic acid (PCA, >99% purity) was purchased from Macklin Biochemical Co., Ltd. (Shanghai, China).

Minimal salt medium (MSM) supplemented with DPrP was used to isolate and culture DPrP-degrading bacterium. MSM was prepared with the following components (per liter): 5.8 g K_2_HPO_4_, 4.5 g KH_2_PO_4_, 2.0 g (NH_4_)_2_SO_4_, 0.34 g MgCl_2_∙6H_2_O, and 1 mL trace element medium stock solution (per liter: 2.6 g CaCl_2_∙2H_2_O, 0.18 g FeSO_4_∙7H_2_O, 0.15 g MnCl_2_∙4H_2_O and 0.24 g Na_2_MoO_4_∙2H_2_O). A Luria-Bertani broth was used to isolate and culture the nondegrading bacterium.

### 2.2. Isolation and Identification of the DBP-Degrading Bacterium and Cooperative Bacterium

After enrichment and selection with 1000 mg/L DPrP, the most efficient strain isolated from agricultural surface layer soils (Hangzhou, Zhejiang Province, China) was selected and designated as ZM05. Strain ZM05 colonies were white, and the morphology of strain ZM0 was G^+^, rod-shaped and motile. The basic criterion for assessing species boundaries is to estimate the genetic relatedness between two genomes [21]. The whole-genome orthologous average nucleotide identity (ANI) values were calculated using an online server: http://enve-omics.ce.gatech.edu/ani/ (accessed on 22 June 2021) [22]. And organisms belonging to the same species typically showing ≥95% ANI among themselves [21]. The results of ANI between strain ZM05 (GenBank Accession No. CP059853.1) and *Glutamicibacter nicotianae* OTC-16 (GenBank Accession No. GCA_003687415.1) was 98.53%. Thus, based on morphological characteristics and ANI calculation of genome, the isolated DBP-degrading bacterium was identified as *Glutamicibacter nicotianae* strain ZM05.

The cooperative bacterium ZM06 was isolated from the same soil samples by using LB plates. Strain ZM06 colonies were white, and the morphology of strain ZM06 was G^−^, rod-shaped and motile. The results of ANI between strain ZM06 (NCBI accession ID: PRJNA739801) and *Acinetobacter tandoii* SE63 (GenBank Accession No. GCA_006965565.1) was 96.25%. Based on morphological characteristics and ANI calculation of genome, the isolated nondegrading bacterium was identified as *Acinetobacter tandoii* strain ZM06.

### 2.3. Monoculture and Coculture Experiments

Early stationary-phase cells of strain ZM05 (OD_600_ = 1.80) or ZM06 (OD_600_ = 1.25) were centrifuged (4 min, 10,000 rpm) and suspended in MSM. Strains ZM05 and ZM06 were inoculated in seventy milliliters of MSM (with or without 0.8 mM CdCl_2_) with 1000 mg/L DPrP to the initial OD_600_ of 0.03, respectively. The monoculture system was inoculated with the same amount of strain ZM05. Coculture and monoculture experiments were conducted in Erlenmeyer flasks with three replicates at 30 °C and 200 rpm.

### 2.4. Sample Preparation and Analysis

Samples were collected to determine the concentrations of DPrP, its metabolites, and Cd(II) during incubation. Extraction and detection of DPrP were carried out according to the method described previously [23]. The metabolites produced during the degradation process were identified by HPLC-MS [24]. The amino acids and fatty acids in the supernatant were detected by LC-MS [25] and GC-MS [26], respectively. The adsorption of Cd(II) on the surface and inside of bacteria was determined by scanning electron microscopy (SEM), transmission electron microscopy (TEM), and energy dispersive spectroscopy (EDS) [27]. The Cd(II) concentration in the supernatant was investigated by ICP-MS (Agilent Technologies 7800 ICP-MS) analysis [28]. Quantitative PCR (qPCR) was used to quantify the ratio of DNA levels of the specific *estG*/*xcpR* genes to determine the ratios of ZM05 to ZM03 in the coculture system under Cd(II) stress [29,30]. The primers used in qPCR are listed in Supplementary Table S1. For the transcriptional analysis, CK (control), CD (monoculture under 0.8 mM Cd^2+^), and CO (coculture under 0.8 mM Cd^2+^) samples were harvested for RNA extraction, and sample information is provided in Table S2.

### 2.5. cDNA Library Construction and RNA-Seq

Total RNA was extracted using the RNeasy Mini Kit (QIAGEN, Hilden, Germany) according to the instructions. RNA quality was monitored on 1% agarose gels, and RNA quantity was measured using Nanodrop One (Thermo Fisher Scientific, Waltham, MA, USA). rRNA was depleted by Ribo-Zero Magnetic Kit (Epicentre) according to the instructions. The RNA samples were subsequently sent to the Guangdong Magigene Biotechnology Co., Ltd. (Guangzhou, China) for library preparation and sequencing. Library preparation was conducted with a NEBNext^®^ Ultra II™ Directional RNA Library Prep Kit (Illumina, San Diego, CA, USA) according to standard protocols. After cluster generation, the library was sequenced on an Illumina HiSeq Xten platform, and 150 bp paired-end reads were generated [31].

### 2.6. RNA-Seq Data Analysis

Raw data in fastq format were processed by Trimmomatic (v.0.36) to acquire clean reads, and clean reads were mapped to NCBI Rfam databases to remove the rRNA sequences by Bowtie2 (v2.33). The remaining mRNA sequences were mapped to the reference genome by Hisat2 (2.1.0) [32]. The HTSeq-count (v0.9.1) was used to obtain the read count and function information of each gene were obtained by HTSeq-count (v0.9.1) according to the mapping results [33]. To make the expression levels of genes comparable among different genes and different experiments, the RPKM was calculated. The read count of each gene was used for differential expression analysis [34]. Differentially expressed genes (DEGs) were identified using edgeR (v3.16.5) [35]. The resulting *p*-value was corrected by Benjamini and Hochberg’s approach for controlling the false discovery rate (FDR). Genes with FDR ≤ 0.05 and | log_2_(fold change) | ≥ 1 were considered candidate DEGs. In addition, those genes were used for KEGG (Kyoto Encyclopedia of Genes and Genomes) enrichment analyses by clusterProfiler (v3.4.4) [36], and gene length bias was corrected. KEGG pathways with FDR ≤ 0.05 were considered significantly enriched. Raw data are deposited in the NCBI (accession ID: PRJNA699047, PRJNA699094, and PRJNA699127).

## 3. Results

### 3.1. Construction of a Synergistic Community under Cd Pressure

**Figure 1:**
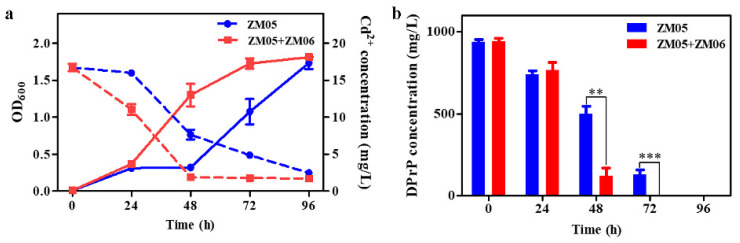
Growth and Cd(II) concentration (**a**) and DPrP degradation (**b**) in monoculture and coculture under Cd(II) contamination. The blue line (column) represents the monoculture of strain ZM05; the red line (column) represents the coculture of strains ZM05 and ZM06. The solid line represents the OD_600_, and the dotted line represents the residual concentration of Cd(II) in the supernatant. All data are presented as the mean ± SE. *** *p* < 0.001, ** *p* < 0.01.

Strain ZM05 completely degraded 1000 mg/L DPrP in 24 h under the optimal conditions (Figure S1), but the growth of strain ZM05 and its degradation activity were significantly inhibited under Cd(II) stress (Figure 1). Through many coculture experiments, it was found that when ZM05 was cocultured with the nondegrading bacterium *Acinetobacter tandoii* ZM06, the residual DPrP and Cd(II) were significantly decreased (Figure 1).

### 3.2. Strain ZM06 Relieved Cd (II) Stress in Coculture

**Figure 2:**
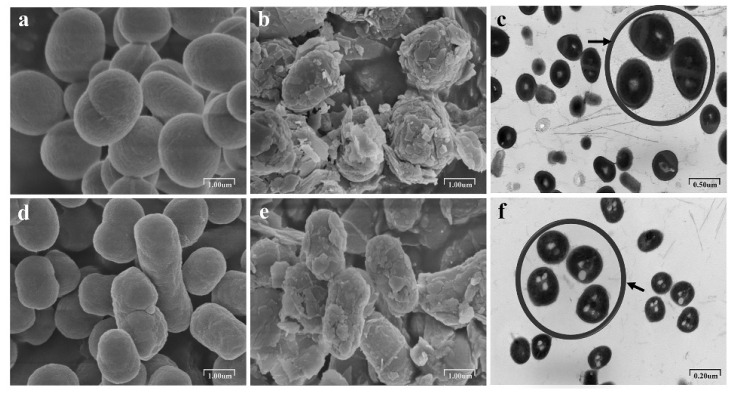
Cell surface morphologies of strains ZM05 (**a**) and ZM06 (**d**) without Cd(II) stress; cell surface morphologies of strains ZM05 (**b**) and ZM06 (**e**) with Cd(II) stress; TEM photographs of strains ZM05 (**c**) and ZM06 (**f**) after adsorption of Cd(II).

The degradation rate of DPrP increased significantly, and the Cd(II) concentration in the supernatant decreased noticeably when strain ZM05 was cocultured with strain ZM06 under Cd(II) stress (Figure 1a). To explore the mechanism of Cd(II) removal, SEM and TEM analyses were carried out. Primarily, SEM analysis was performed to compare the cell surface morphology before and after adsorption to determine whether ZM06 could alleviate the cytotoxicity caused by Cd(II) stress. As shown in Figure 2a,d, the surfaces of strains ZM05 and ZM06 were smooth and clear without adhesion before Cd(II) stress, while the morphologies of ZM05 and ZM06 changed significantly under Cd(II) stress, and many flaky substances, which were speculated to be the “microprecipitation” of inorganic salts, were adsorbed on the cell surface (Figure 2b,e).

To identify whether ZM05 and ZM06 reduce the free Cd(II) in the culture medium by absorbing Cd(II) into cells through active transport, TEM analysis and EDS analysis were used. There were obvious vacuoles in both ZM05 and ZM06 cells (Figure 2c,f), which indicated that the cells had stress responses to Cd(II) stress. However, EDS of the cytoplasm of ZM05 and ZM06 cells showed that neither ZM05 nor ZM06 accumulate Cd(II) or form any cadmium-containing phosphate granules intracellularly (Figure S2).

### 3.3. Strain ZM06 Utilized the Metabolites of DPrP

**Figure 3:**
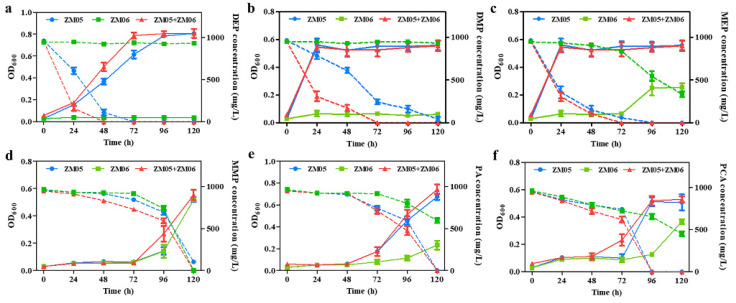
Growth and degradation characteristics of ZM05 (blue), ZM06 (green), and coculture (red) of ZM05 and ZM06 when supplemented with DEP (**a**), DMP (**b**), MEP (**c**), MMP (**d**), PA (**e**) or PCA (**f**) as the sole carbon source under Cd(II) contamination. The solid line represents the OD_600_, and the dotted line represents the residual concentration of the carbon source in the medium. All data are shown as the mean ± SE.

To detect the growth of strain ZM06 in the coculture system, qPCR was performed with specific primers. The nondegrading bacterium ZM06 was present in high proportions during the logarithmic growth period (Figure S3). To explore the survival mechanism of strain ZM06 in the coculture system and the interaction mechanism between strains ZM05 and ZM06 under Cd(II) stress, monoculture and coculture experiments supplied with DPrP or its metabolites (DEP, DMP, MEP, MMP, PA, or PCA) were performed. The detection of growth ability and substrates metabolic ability in mono- and coculture showed that ZM06 could utilize the downstream intermediates of DPrP produced by ZM05 as carbon source, including MEP, MMP, PA, and PCA (Figure 3). In particular, the degradation efficiency of DEP and DMP in the coculture system was significantly higher than those in the monoculture of strain ZM05.

### 3.4. Transcriptional Response of Strain ZM05 to Cd(II) Stress in Monoculture

**Figure 4:**
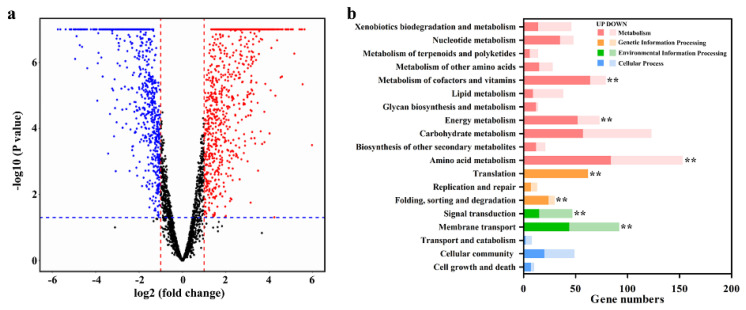
Volcano plot (**a**) and KEGG enrichment analysis (**b**) of DEGs from strain ZM05 with or without Cd(II) stress. ** *p* < 0.01.

To analyze the impacts of Cd(II) on the gene expression of strain ZM05 during DPrP degradation, transcriptional differences between strain ZM05 with and without Cd(II) stress in monoculture systems were determined. The results of the comparative transcriptomic analysis revealed 579 significantly upregulated genes and 439 significantly downregulated genes in strain ZM05 under Cd(II) stress (Figure 4a). The DEGs of strain ZM05 without Cd(II) stress vs. ZM05 with Cd(II) stress were significantly identified in “metabolism”, “genetic information processing” and “environmental information processing” (Figure 4b). Upregulated DEGs related to ‘metabolism of cofactors and vitamins’, ‘energy metabolism’, ‘amino acid metabolism’, ‘translation’ and ‘folding, sorting and degradation’ outnumbered downregulated DEGs. A converse trend was obtained in ‘signal transduction’ and ‘membrane transport’, in which downregulated DEGs outnumbered upregulated DEGs. As shown in Figure S4a, genes involved in quorum sensing, ABC transporters, two-component systems, pyruvate metabolism, fatty acid degradation, and benzoate degradation were downregulated, while genes involved in the ribosome, oxidative phosphorylation, purine metabolism, and the citrate cycle were upregulated when strain ZM05 was grown under Cd(II) stress. These results indicated that Cd(II) stress inhibited the normal activity of strain ZM05, which was followed by a stress response.

### 3.5. Gene Expression of Strain ZM05 in Coculture and Monoculture

**Figure 5:**
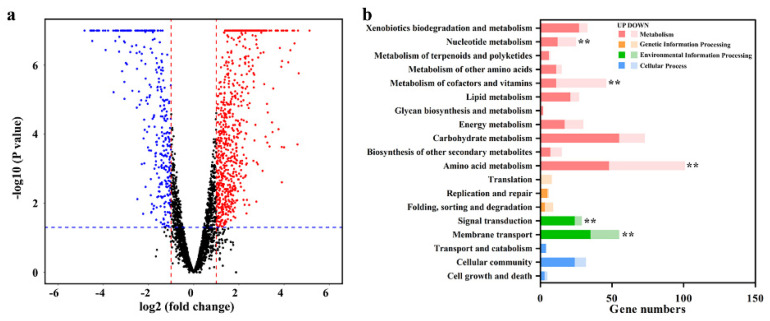
Volcano plot (**a**) and KEGG enrichment analysis (**b**) of DEGs from strain ZM05 in the monoculture system or coculture system under Cd(II) stress. ** *p* < 0.01.

In light of the DPrP degradation behavior (Figure S1), strain ZM06 synergistic promoted DPrP degradation by strain ZM05 when cultured under Cd(II) stress but had no such effect when grown without Cd(II) stress. Hence, we assumed that under Cd(II) stress, strain ZM06 could affect the gene expression of strain ZM05 in coculture systems. The volcano map reveals that strain ZM06 influenced the gene expression of strain ZM05 in the coculture system under Cd(II) stress, which represented 343 upregulated genes and 212 downregulated genes (Figure 5a). The significant DEGs of strain ZM05 in the monoculture system vs. ZM05 in the coculture system were mainly identified in “metabolism” and “environmental information processing” (Figure 5b). The upregulated DEGs related to ‘signal transduction’, ‘amino acid metabolism’ and ‘membrane transport’ outnumbered the downregulated DEGs significantly, while the upregulated DEGs related to ‘nucleotide metabolism’ and ‘metabolism of cofactors and vitamins’ were significantly fewer than the downregulated DEGs. As shown in Figure S4b, the depleted KEGG pathways from strain ZM05 included ribosome and valine, leucine, and isoleucine biosynthesis; other KEGG pathways, including quorum sensing, ABC transporters, two-component system, glycolysis, pyruvate metabolism, and benzoate degradation, were upregulated.

## 4. Discussion

The coexistence of cadmium with DPrP in the environment is a particular challenge for bioremediation because cadmium is toxic and cannot be degraded by biological processes [37]. However, the DPrP-degrading bacterium ZM05 shows weak resistance to Cd(II) stress, with a low rate of growth and DPrP degradation. To address environmental pressure, the synergistic relationships among microbes can be advantageous [38,39]. According to the analysis of DPrP degradation, cooperative interaction between strains ZM05 and ZM06 forms when straining ZM05 experiences Cd(II) stress.

### 4.1. Cd (II) Strongly Stressed Strain ZM05

Cadmium, one of the most toxic heavy metals, can disrupt cell proliferation and differentiation, cell cycle progression, apoptosis, and other cellular activities by proteotoxicity and DNA damage induced by oxidative stress [40,41]. Furthermore, microorganisms can alter their structure, physiology, and metabolic ability in detrimental environments [42]. In this study, SEM results revealed that the morphology of strain ZM05 was significantly changed, and bacteria formed insoluble cadmium salts under Cd(II) stress. Changes in the morphology of microorganisms after heavy metal biosorption generate an adaptive mechanism for surviving the stress forced by heavy metals [43]. As reported previously, Cd(II) stress clearly caused membrane indentations and decreased the surface area/volume ratio of *Acidiphilium symbioticum* H8, resulting in more elongated cells [44].

In response to Cd(II) stress, strain ZM05 upregulates genes involved in a stress response such as cold shock protein (*cspA*), riboflavin synthesis genes (*rib*), and thioredoxin reductase (*trxB*, *TRR*) (Table S3). Cold shock proteins play important roles in transcription, mRNA stability, and translation [45], and riboflavin, as an important coenzyme of oxidoreductases, prevents oxidative stress [46]. Thioredoxin also serves as a stress-response factor in some bacteria [47]. The upregulation of genes involved in the stress response suggests that Cd(II) causes strong environmental stress on strain ZM05. Interestingly, the expression of the ribosome was significantly upregulated, suggesting that the ribosome probably plays an important role in the repair of cadmium-mediated cellular damage [48].

### 4.2. Strain ZM06 Accelerated the Degradation of DPrP

Many degrading bacteria are closely related to other microbes, and they constitute composite microbial degradation systems with improved capability [49], especially in an unfavorable environment, microbes prefer to form consortia to resist stress [15]. In this study, both strains ZM05 and ZM06 adsorbed cadmium on the surface and did not transport Cd(II) into cells. The addition of strain ZM06 greatly reduced the concentration of free cadmium in the culture system. The results illustrated that the nondegrading bacterium ZM06 relieved the stress of Cd(II) on the degrading bacterium ZM05.

Simultaneously, microbes can cooperate with the bacteria that metabolize the downstream metabolites to form bacterial consortia to accelerate the degradation of organic pollutants [50,51]. Under Cd(II) stress, the accelerated degradation of DPrP was presumable attributed to faster depletion of downstream intermediates by strain ZM06 in the coculture system (Figure 3). Strain ZM05 downregulated the genes responsible for the upstream metabolism of DPrP degradation but upregulated the genes responsible for the downstream metabolism in the monoculture system under Cd(II) stress, which indicated that stress impeded the committed step during degradation (Table S4). When cocultured with strain ZM06, the variations in the transcriptional levels of the genes involved in DPrP degradation occurred in the opposite directions of the variations in the monoculture. The utilization of DPrP downstream metabolites by strain ZM06 not only relieved the inhibition of metabolites but also accelerated the key degradation steps of strain ZM05. Reducing the accumulation of intermediates and thus accelerating the consumption of substrates that produce growth-inhibiting intermediates, is important to optimize the desired biotransformation [52].

### 4.3. Strain ZM06 Contributed Amino Acids and Fatty Acids to Strain ZM05

In cooperative relationships of microbes, the exchange of substances occurs frequently [53]. Under Cd(II) stress, monoculture of strain ZM05 upregulated valine, leucine, and isoleucine biosynthesis, while coculture with ZM06 downregulated valine, leucine, and isoleucine biosynthesis (Figure S4). At the same time, strain ZM05 upregulated the genes involved in translation in monoculture but downregulated the genes involved in translation when cocultured with strain ZM06 (Figure 4b and Figure 5b). Moreover, the concentration of leucine in coculture was significantly higher than that in monoculture (Figure S6b). The results indicated that strain ZM06 probably offers amino acids and proteins to strain ZM05 under Cd(II) stress.

Similarly, Cd(II) stress caused the downregulation of fatty acid degradation required for energy production in strain ZM05 in the monoculture system. Strain ZM05 upregulated fatty acid degradation in the coculture system (Figure S4). And the detected palmitic acid was more abundant in the coculture system (Figure S5b). We suggest that strain ZM06 may contribute fatty acids to support the response of strain ZM05 to Cd(II) stress. Bacterial communities can make efficient use of limited resources through metabolites exchange, providing survival advantages under challenging conditions [54]. Strain ZM06 effectively alleviated Cd(II) stress on ZM05, and much substance and signal communication occurred between strains ZM05 and ZM06.

### 4.4. Mechanism of Cooperation between Strain ZM05 and Strain ZM06

**Figure 6:**
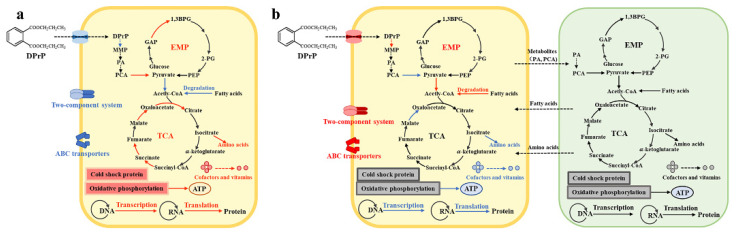
Schematic mechanism of strain ZM05 under Cd(II) stress (**a**) and the interactions between strain ZM05 and strain ZM06 (**b**)during DPrP biodegradation. Yellow oblong represents strain ZM05; green oblong represents strain ZM06. The red text represents upregulated metabolism; blue text represents downregulated metabolism.

According to the above results, we construct a model of the interaction between the DPrP-degrading bacterium ZM05 and the nondegrading bacterium ZM06, as shown in Figure 6. In a coculture system, DPrP was degraded by strain ZM05, and readily available intermediates were utilized not only by strain ZM05 but also by strain ZM06 as a carbon source. The stress of Cd(II) leads to significant changes in the expression of genes involved in the crucial step of DPrP degradation, signal transduction, and energy metabolism. The symbiotic bacterium ZM06, surviving on the metabolites of DPrP, supplies amino acids and fatty acids to strain ZM05 to coping with stress. At the same time, the coculture strengthened the signaling and substrate communication between the two bacteria, and the coculture system is stable and efficiently degrades DPrP.

## 5. Conclusions

In summary, this study proves that a consortium of the DPrP-degrading bacterium ZM05 and the nondegrading bacterium ZM06 has an improved performance in degrading DPrP under Cd(II) stress. Strain ZM06 could not degrade DPrP, but it offers fatty acids and amino acids to strain ZM05 in response in the coculture system, which might relieve the pressure on strain ZM05. Our results clarify the interactions within the synergistic community during DPrP degradation under Cd(II) stress and hint at environmental pressure as a major driver of species co-occurrence. Moreover, this study provides new insights and theoretical basis for the application of microbial consortium in the remediation of pollutants in an unfavorable environment.

## Supplementary Materials

The following are available online at https://www.mdpi.com/article/10.3390/microorganisms9071417/s1, Table S1: Primers used for quantitative PCR, Table S2: The information for the transcriptomic sample; Table S3: Gene involved in the stress response of strain ZM05 annotated by KEGG database; Table S4: Degradation gene of strain ZM05 annotated by KEGG database; Figure S1: Growth curve and DPrP degradation behavior in monoculture and coculture under optimum conditions; Figure S2: EDS analysis of strain ZM05 and ZM06 under Cd (II) stress; Figure S3: Relative abundances of strain ZM05 and strain ZM06 in coculture system under Cd (II) stress; Figure S4: KEGG analyses of DEGs from strain ZM05 with or without Cd (II) stress and strain ZM05 in the mono-culture system or coculture system under Cd (II) stress; Figure S5. Amino acids and fatty acids in monoculture and coculture systems under Cd (II) stress.
