# Supplementary material for: Acinetobacter tandoii ZM06 Assists Glutamicibacter nicotianae ZM05 in Resisting Cadmium Pressure to Preserve Dipropyl Phthalate Biodegradation
Source: Microorganisms. 2021 Jun 30;9(7):1417. doi: 10.3390/microorganisms9071417 (PMC8307640; doi:10.3390/microorganisms9071417)
Supplement: Supplementary file 1 [file microorganisms-09-01417-s001.zip › microorganisms-1250737-SI.pdf]

Table S1 Primers used for quantitative PCR.

Name	Sequence (5'-3')
estG FP	CTTTTGTCCTCCAGGCTCC
estG RP	GCGCGAGGCGATATTCAAAGC
xcpR FP	GCGATAGCAGGGACTAATGC
xcpR RP	TCTGCTGAAGGCACATCAAG

Table S2 The information for the transcriptomic sample.

Nam	Detailed description	Sampling time
CK	Mono-culture of ZM05 with DPrP as the sole carbon source	18h
CD	Mono-culture of ZM05 with DPrP as the sole carbon source under Cd (II) stress	48 h
CO	Coculture of ZM05 and ZM06 with DPrP as the sole carbon source under Cd (II) stress	48 h

Table S3 Gene involved in the stress response of strain ZM05 annotated by KEGG database

**Gene**	**Gene annotation**	**CD vs. CK**	**CO vs. CD**
cspA	cold shock protein	2.03	-
trxB,TRR	thioredoxin reductase (NADPH)	2.1	-
ribH	Riboflavin synthase, alpha subunit	2.62	-
ribE	Riboflavin synthase, alpha subunit	2.36	-

"-" means no significant change.

Table S4 Degradation gene of strain ZM05 annotated by KEGG database

Gene	Gene annotation	CD vs. CK	CO vs. CD
catE	catechol 2,3-dioxygenase	-1.16	1.09
catA	catechol 1,2-dioxygenase	-2.65	2.02
yvaK	carboxylesterase	-0.01	0.05
K06999	carboxylesterase	-2.83	1.31
pcaC	4-carboxymuconolactone decarboxylase	3.63	-1.90
pcaD	3-oxoadipate enol-lactonase	3.36	-1.31
pcaB	3-carboxy-cis,cis-muconate cycloisomerase	3.17	-0.79
pcaG	protocatechuate 3,4-dioxygenase, alpha subunit	4.78	-0.72
pcaH	protocatechuate 3,4-dioxygenase, beta subunit	4.93	-0.67
pobA	p-hydroxybenzoate 3-monooxygenase	4.75	-1.89
pcaI	3-oxoadipate CoA-transferase	2.94	-3.08
pcaJ	pca regulon regulatory protein	3.14	-3.44
pcaR	pca regulon regulatory protein	2.52	-2.61

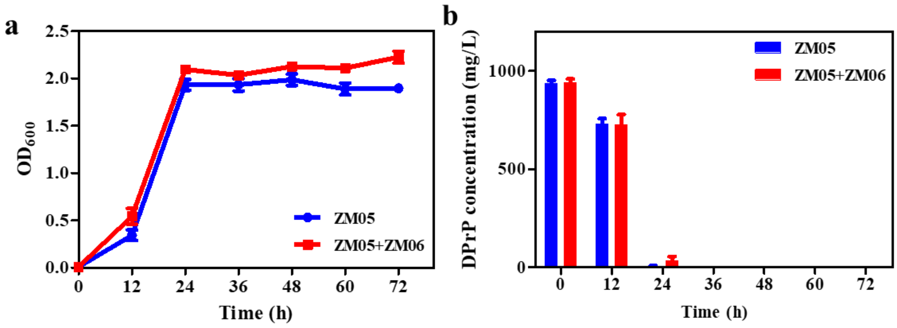

Fig. S1. Growth curve (**a**) and DPrP degradation behavior (**b**) in monoculture and coculture under optimum conditions. All data are presented as the mean ± SE. The blue line (column) represents the monoculture of strain ZM05; the red line (column) represents the coculture of strains ZM05 and ZM06. ***p<0.001, **p<0.01, *p<0.05.

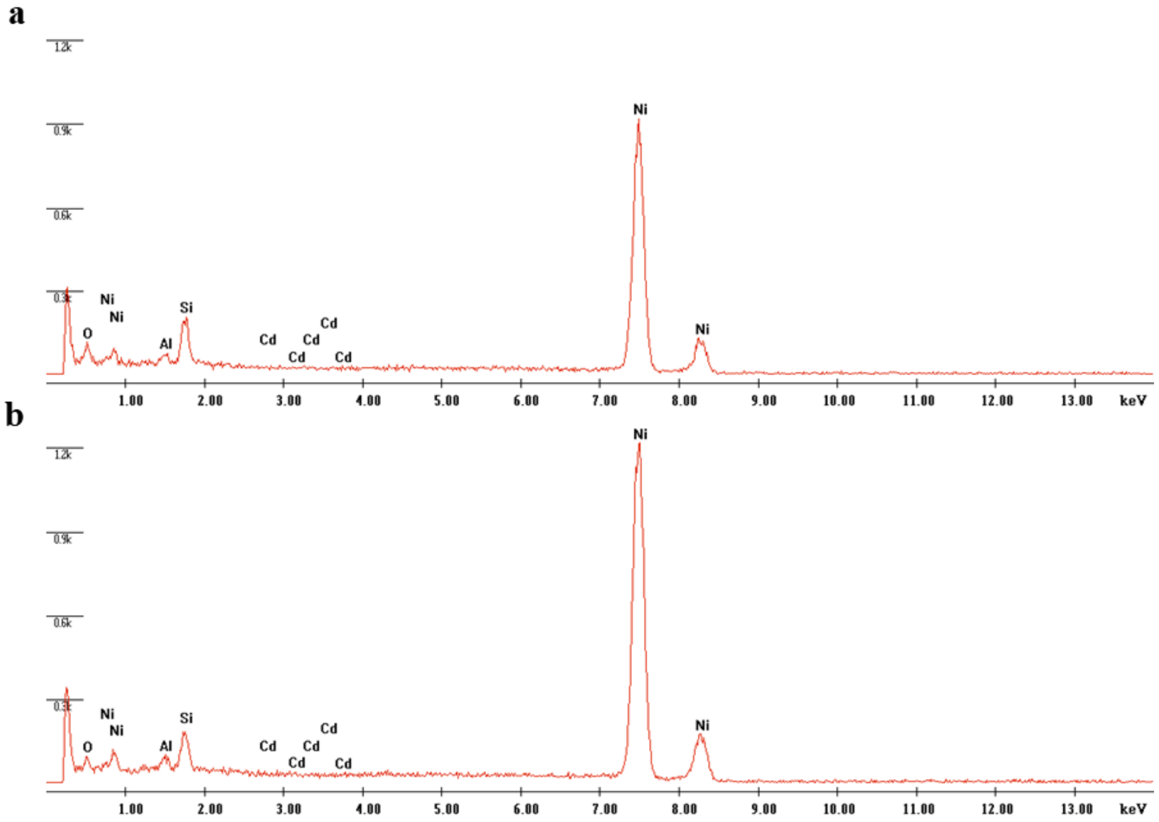

Fig.S2. Energy dispersive spectrum (EDS) analysis of strain ZM05 (a) and ZM06 (b) under Cd (II) stress.

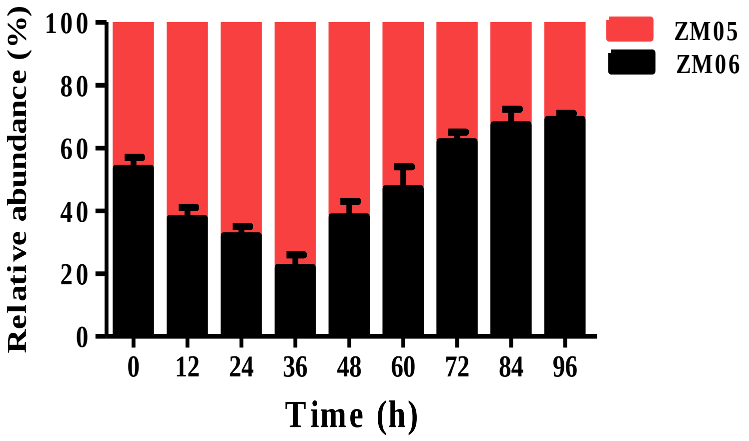

Fig.S3. Relative abundances of strain ZM05 and strain ZM06 in coculture system under Cd (II) stress. All data are presented as the mean ± SE.

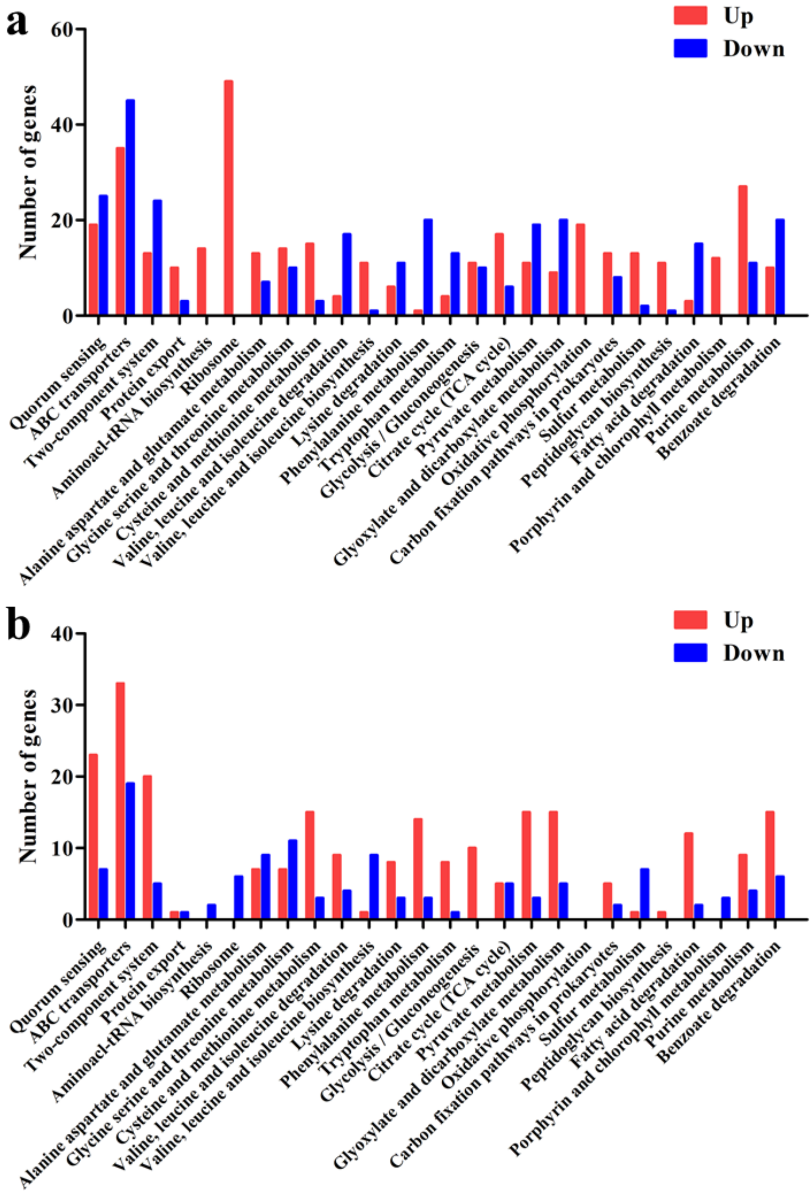

Fig. S4. Metabolism pathway (KEGG) analyses of DEGs from strain ZM05 with or without Cd (II) stress (**a**) and strain ZM05 in mono-culture system or coculture system under Cd (II) stress (**b**).
